# Evaluation of association of maternal IL-10 polymorphisms with risk of preeclampsia by A meta-analysis

**DOI:** 10.1111/jcmm.12434

**Published:** 2014-09-25

**Authors:** Wenchao Yang, Zhenmin Zhu, Jin Wang, Wei Ye, Yong Ding

**Affiliations:** a Management Department, Shanghai Medical Instrumentation CollegeShanghai, China; b Medical Electronic Information Department, Shanghai Medical Instrumentation CollegeShanghai, China

**Keywords:** IL-10, polymorphism, preeclampsia, meta-analysis

## Abstract

Emerging evidence shows that interleukin (IL)-10 gene polymorphisms can regulate its expression level and thus influence person's susceptibility to preeclampsia. However, various published results were inconsistent. To explore the association between maternal IL-10 gene polymorphisms and preeclampsia, we performed a meta-analysis based upon 11 individual studies here. Our meta-analysis results indicated that IL-10 -819C/T (C *versus* T, OR = 1.28, 95% CI = 1.08–1.50, *P* = 0.003) and -592C/A (C *versus* A, OR = 1.28, 95% CI = 1.03–1.59, *P* = 0.03) polymorphisms were associated with preeclampsia. Although there was no overall association between -1082A/G polymorphism and preeclampsia (G *versus* A, OR = 0.93, 95% CI = 0.77–1.13, *P* = 0.49), such association existed among Asian (G *versus* A, OR = 1.29, 95% CI = 1.04–1.60, *P* = 0.02) and South American (G *versus* A, OR = 0.72, 95% CI = 0.54–0.94, *P* = 0.02) populations in the subgroup analysis stratified by continents.

## Introduction

Preeclampsia is a common pregnancy-specific disorder characterized by new-onset hypertension in combination with proteinuria after 20 weeks of gestation [1]. It occurs in about 2–8% pregnancies and has become one of the three leading causes of maternal and neonatal morbidity and mortality [2,3]. Although the precise aetiology of preeclampsia is unknown because of its heterogeneous origins, the immune system has been found to play a significant role in the development of preeclampsia [4].

Normal pregnancy entails the shift of Th1/Th2 ratio towards Th2-type reactions [5,6]. Th1-type cytokines are responsible for several cell-mediated cytotoxic and inflammatory reactions and can produce a proinflammatory milieu. Excessive Th1-type cytokines like interleukin (IL)-2, tumour necrosis factor-alpha (TNF-α) and interferon-gamma (IFN-γ) have been reported to be detrimental to foetus and associated with preeclampsia [6–9]. Th2 cells are involved in the regulation of Th1 cell development and the maintenance of an anti-inflammatory environment besides the common antibody responses [5,10,11]. As foetus is like a semi-allograft, the ability of Th2 cells to protect against allograft rejection plays a vital role during pregnancy [5,10].

Interleukin-10, originally described as a crucial Th2-type cytokine because of its anti-inflammatory actions, plays a pivotal role in pregnancy maintenance and development [12,13]. It can help to establish the Th2 immune environment and inhibit the secretion of Th1-type cytokines like IL-6, TNF-α and IFN-gamma [14–16]. Also, IL-10 has been reported to contribute to trophoblast invasion, corpus luteum maturation and placental angiogenesis during pregnancy [12,15,17–20]. Evidence showed that the expression levels of placental and decidual IL-10 were altered in preeclampsia patients [21–23]. Reduced production of IL-10 has been suggested to cause a proinflammatory cytokine response and thus lead to the pathogenesis of preeclampsia [24,25].

As polymorphisms in regulatory regions of cytokine genes can influence their expression levels, they may be related to person's predisposition to certain diseases [26]. Given the recognized importance of IL-10 in pregnancy, several polymorphic sites in its regulatory regions including -1082A/G (rs1800896), -819C/T (rs1800871) and -592C/A (rs1800872) have been widely investigated for their potential correlation with preeclampsia because of their reported capability of altering the gene expression level of IL-10[27–30]. However, the results are still inconclusive or controversial. Although three meta-analyses have suggested that the -1082A/G polymorphism of the IL-10 gene was not associated with preeclampsia [31–33], the latest study still supported such association [15]. Although Daher *et al*. have pointed out that such association could be subject to ethnicity, no related meta-analysis based upon a specific ethnicity has been reported so far [34]. Besides, there is still a lack of meta-analysis of other IL-10 polymorphic sites' association with preeclampsia. To address these issues, we performed a systemic review and a meta-analysis of all genetic association studies of maternal IL-10 polymorphisms related to preeclampsia to investigate the association between maternal IL-10 polymorphisms and preeclampsia. This systemic review may help to enhance our understanding of the role of IL-10 in the aetiology of preeclampsia and early identification of persons predisposed to preeclampsia.

## Materials and methods

### Literature search

A systematic literature search of PubMed, Web of Science and Scopus databases was conducted by two researchers independently for all relevant articles published before March 2014. The research key words included ‘pregnancy induced hypertension’, ‘gestational hypertension’, ‘preeclampsia’, ‘genotype’, ‘SNP’, ‘mutation’, ‘polymorphism’ ‘IL-10’ and ‘interleukin 10’.

### Inclusion and exclusion criteria

Studies included for this meta-analysis should meet the following criteria: (*i*) case–control studies or cohort studies focusing on the association between IL-10 polymorphism and preeclampsia; (*ii*) patients have been clinically diagnosed with preeclampsia and preeclampsia was defined as hypertension (≥140/90 mm Hg on two occasions ≥6 hrs apart) with proteinuria (>300 mg/24 hrs or ≥1+ dipstick in urine sample) after 20 weeks of gestation; (*iii*) the numbers of patients and normotensive pregnant women with various genotypes were available. The exclusion criteria of the meta-analysis were: (*i*) animal studies; (*ii*) meta-analyses, reviews, meeting abstracts or editorial comments; (*iii*) studies with duplicate data or incomplete data for odds ratio (OR) calculation; (*iv*) IL-10 polymorphic sites reported only once.

### Data extraction

Information was extracted from all eligible studies by two authors independently and checked by a third author with disparities resolved by consensus. The collected data included the first author's name, publication date, region/ethnicity, genotyping method and the total number of cases and controls.

### Statistical analysis

We used Review Manager 5.2 (Cochrane Collaboration, Oxford, UK) and Stata (Version 12.0; Stata Corporation, College Station TX, USA) for all the statistical analysis. The association was evaluated with the use of the allelic model (mutation [M] allele *versus* wild [W] allele), the dominant model (WM+MM *versus* WW), the recessive model (MM *versus* WM+WW) and the co-dominant model (WM *versus* WW+MM) respectively. We calculated the OR and 95% CI for each study as well as the combined OR and corresponding 95% CI for all the included studies. The heterogeneity between individual studies was assessed using chi-squared-based Q-tests with the significance level set at *P* < 0.1. If the heterogeneity existed among the included studies, we calculated the pooled OR using the random-effect model (the DerSimonian and Laird method). Otherwise, we adopted the fixed-effect model (the Mantel–Haenszel method). The significance of the pooled OR was assessed by *Z*-test with *P* < 0.1 considered significant.

For each study, the Hardy–Weinberg equilibrium (HWE) was assessed by Fisher's exact test with *P* < 0.05 considered significant. In every pooled analysis, studies with controls not in HWE were still considered but the corresponding sensitivity analysis without these studies was also performed. The potential publication bias was checked by Begg's funnel plot and the funnel plot asymmetry was assessed by Egger's linear regression test with the significance level set at *P* < 0.05.

## Results

### Literature selection

We found a total of 375 articles after an initial search from the PubMed, Web of Science and Scopus databases. By reviewing the titles and abstracts, 355 of them were excluded because of no relevance to the association of IL-10 polymorphisms with preeclampsia. After excluding reviews, meta-analyses, studies without sufficient data or replication study, 12 eligible studies were finally included in this meta-analysis [6,7,15,24,34–41]. A total of 1861 preeclampsia patients and 3632 normotensive pregnant women were included in this study.

Table 1 summarized the characteristics of 12 included studies. There were 10 case–control studies [6,7,15,24,34–39] and 1 cohort study [40] involving IL-10 -1082A/G polymorphism, 5 case–control studies involving IL-10 -819C/T polymorphism [6,7,37,38,41] and 3 case–control studies involving IL-10 -592C/A polymorphism [6,7,38]. Only one study investigated the association of IL-10 -2849G/A polymorphism with preeclampsia [42].

**Table 1:** Characteristics of the 12 eligible studies included for the investigation of IL-10 polymorphisms' association with preeclampsia

First author	Year	Country/Continent	Ethnicity	Sample size (preeclampsia/control)	SNP studied	Method
Stonek [35]	2008a	Austria/Europe	Caucasian	107/107	-1082A/G	Microarray
Valencia Villalvazo [36]	2012	Mexico/the Americas	Mexican-Mestizo, Maya-Mestizo	411/613	-1082A/G	TaqMan technology
Haggerty [37]	2005	USA/the Americas	Black, White	150/661	-1082A/G, -819C/T	TaqMan technology
Mirahmadian [6]	2008	Iran/Asia	Asian	160/100	-1082A/G, -819C/T, -592C/A	PCR-SSP
Kamali-Sarvestani [7]	2006	Iran/Asia	Asian	134/164	-1082A/G, -819C/T, -592C/A	PCR-SSP (-1082A/G), PCR-RFLP(-819 C/T, -592C/A)
de Lima [38]	2009	Brazil/the Americas	Mulatto	165/101	-1082A/G, -819C/T, -592C/A	PCR-SSP
Daher [34]	2006	Brazil/the Americas	Black, White, Mulatto	151/189	-1082A/G	PCR-SSP
Sowmya [39]	2013	India/Asia	Asian	88/100	-1082A/G	PCR-SSP
Stonek [40]	2008b	Austria/Europe	Caucasian	254/1362	-1082A/G	Microarray
Elhawary [15]	2013	Egypt/Africa	African	20/20	-1082A/G	PCR-RFLP
Sowmya [41]	2014	India/Asia	Asian	120/120	-819C/T	PCR-SSP
Vural [24]	2010	Turkey/Europe-Asia	European-Asian	101/95	-1082A/G	PCR-SSP

### Association between IL-10 -1082A/G polymorphism and preeclampsia

**Fig. 1:**
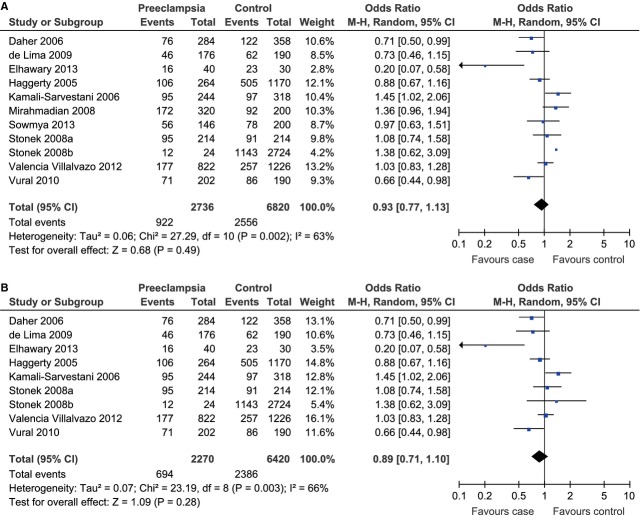
Forest plot of preeclampsia associated with IL-10 -1082A/G polymorphism under the allelic model (G allele *versus* A allele) (**A**) and the corresponding sensitivity analysis with the exclusion of studies not in HWE (**B**).

A total of 1368 cases and 3410 controls from 10 case–control studies and 1 cohort study were included for the evaluation. Significant heterogeneity existed and therefore the random-effect model was adopted to pool the results (*P*_heterogeneity_ = 0.002, *I*^2^ = 63%). The meta-analysis result showed that IL-10 -1082A/G polymorphism was not associated with the risk of preeclampsia under the allelic model (G allele *versus* A allele, OR = 0.93, 95% CI = 0.77–1.13, *P* = 0.49; Fig. 1A). As there were two studies with controls not in HWE [6,39], we conducted a sensitivity analysis with these two studies excluded and the result still indicated that there was a lack of association between IL-10 -1082A/G polymorphism and the risk of preeclampsia (G allele *versus* A allele, OR = 0.89, 95% CI = 0.71–1.10, *P* = 0.28; Fig. 1B).

**Fig. 2:**
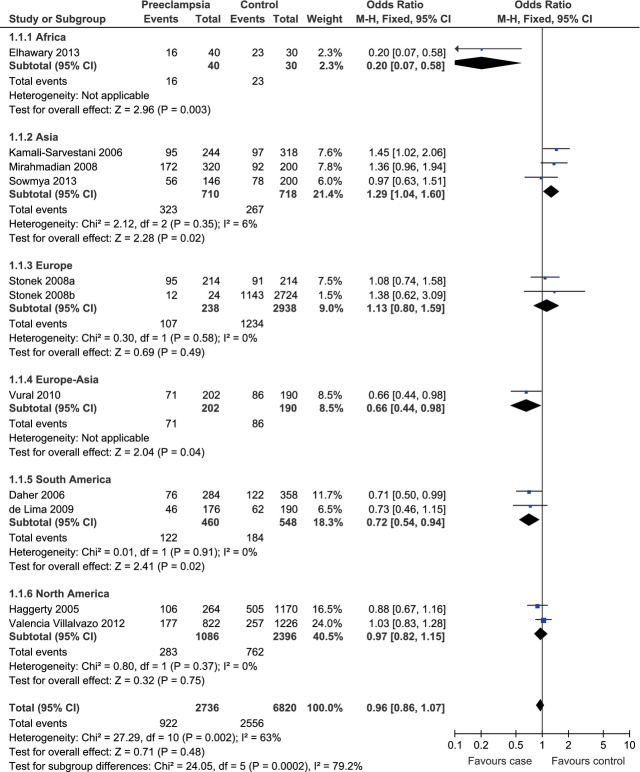
Forest plot of preeclampsia associated with IL-10 -1082A/G polymorphism stratified by continents under the allelic model (G allele *versus* A allele). The Europe-Asia subgroup included countries spanning Europe and Asia.

**Fig. 3:**
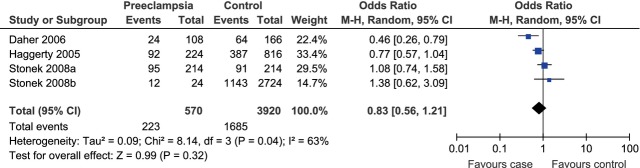
Forest plot of the risk of preeclampsia among white women associated with IL-10 -1082A/G polymorphism under the allelic model (G allele *versus* A allele).

In the subgroup analysis stratified by continents, we found that the heterogeneity was significantly lower in the Asia (*P*_heterogeneity_ = 0.35, *I*^2^ = 6%), Europe (*P*_heterogeneity_ = 0.58, *I*^2^ = 0%), South America (*P*_heterogeneity_ = 0.91, *I*^2^ = 0%) and North America groups (*P*_heterogeneity_ = 0.37, *I*^2^ = 0%) than in the whole population (*P*_heterogeneity_ = 0.002, *I*^2^ = 63%; Fig. 2). Therefore, the regions where the individual studies were performed could explain the source of heterogeneity. This result was somewhat reminiscent of Daher *et al*.'s previous report that the association between IL-10 -1082A/G polymorphism and preeclampsia were only observed in white women instead of non-white women [34]. However, our meta-analysis results based upon four previously published relevant studies did not support the association between IL-10 -1082A/G polymorphism and the risk of preeclampsia among white women under the allelic model (G allele *versus* A allele, OR = 0.83, 95% CI = 0.56–1.21, *P* = 0.32; Fig. 3).

**Fig. 4:**
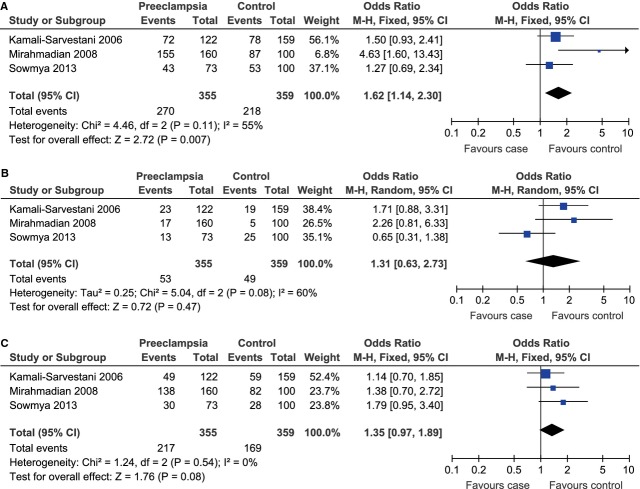
Forest plot of the risk of preeclampsia among Asian population associated with IL-10 -1082A/G polymorphism under the dominant (GG+GA *versus* AA) (**A**), recessive (GG *versus* GA+AA) (**B**) and co-dominant (GA *versus* GG+AA) (**C**) model.

**Fig. 5:**
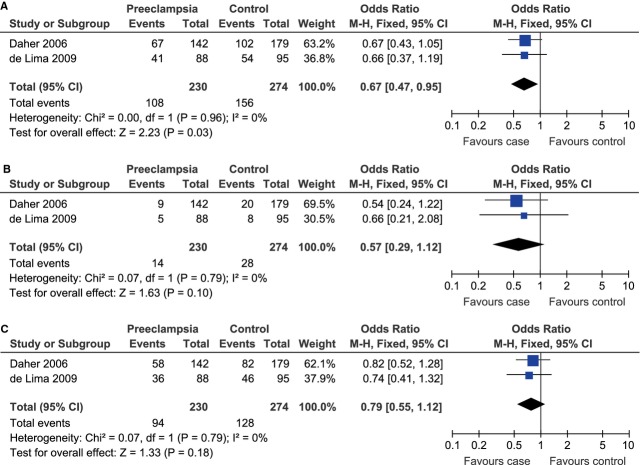
Forest plot of the risk of preeclampsia among South American population associated with IL-10 -1082A/G polymorphism under the dominant (GG+GA *versus* AA) (**A**), recessive (GG *versus* GA+AA) (**B**) and co-dominant (GA *versus* GG+AA) (**C**) model.

Besides, we observed a significant association between IL-10 -1082A/G polymorphism and the risk of preeclampsia in the Asia (G allele *versus* A allele, OR = 1.29, 95% CI = 1.04–1.60, *P* = 0.02) and the South America (G allele *versus* A allele, OR = 0.72, 95% CI = 0.54–0.94, *P* = 0.02) subgroups under the allelic model. However, under the allelic model (G allele *versus* A allele), there was no such association in the Europe (OR = 1.13, 95% CI = 0.80–1.59, *P* = 0.49) or the North America group (OR = 0.97, 95% CI = 0.82–1.15, *P* = 0.75; Fig. 2). To further explore the potential way the G allele affected the risk of preeclampsia among Asian and South American populations, we then evaluated the association under other three genetic models. For Asian population, as there was no obvious heterogeneity under the dominant (GG+GA *versus* AA, *P*_heterogeneity_ = 0.11, *I*^2^ = 55%) or co-dominant model (GA *versus* GG+AA, *P*_heterogeneity_ = 0.54, *I*^2^ = 0%), the fixed-effect model was used for these two genetic models. The random-effect model was used for the recessive model (GG *versus* GA+AA) because of the existence of significant heterogeneity (*P*_heterogeneity_ = 0.08, *I*^2^ = 60%). Under the dominant model, the pooled data indicated that the GG and GA phenotypes were linked to the increased risk of preeclampsia among Asian population (OR = 1.62, 95% CI = 1.14–2.30, *P* = 0.007; Fig. 4A). There was no evidence for similar association under the recessive (OR = 1.31, 95% CI = 0.63–2.73, *P* = 0.47; Fig. 4B) or co-dominant model (OR = 1.35, 95% CI = 0.97–1.89, *P* = 0.08; Fig. 4C). As for South American population, the fixed-effect model was used for the dominant (*P*_heterogeneity_ = 0.96, *I*^2^ = 0%), recessive (*P*_heterogeneity_ = 0.79, *I*^2^ = 0%) and co-dominant (*P*_heterogeneity_ = 0.79, *I*^2^ = 0%) models because of lack of between-study heterogeneity. Under the dominant model, the pooled data indicated that the GG and GA phenotypes were linked to the decreased risk of preeclampsia among South American population (OR = 0.67, 95% CI = 0.47–0.95, *P* = 0.03; Fig. 5A). There was no evidence for similar association under the recessive (OR = 0.57, 95% CI = 0.29–1.12, *P* = 0.10; Fig. 5B) or co-dominant model (OR = 0.79, 95% CI = 0.55–1.12, *P* = 0.18; Fig. 5C).

### Association between IL-10 -819C/T polymorphism and preeclampsia

**Fig. 6:**
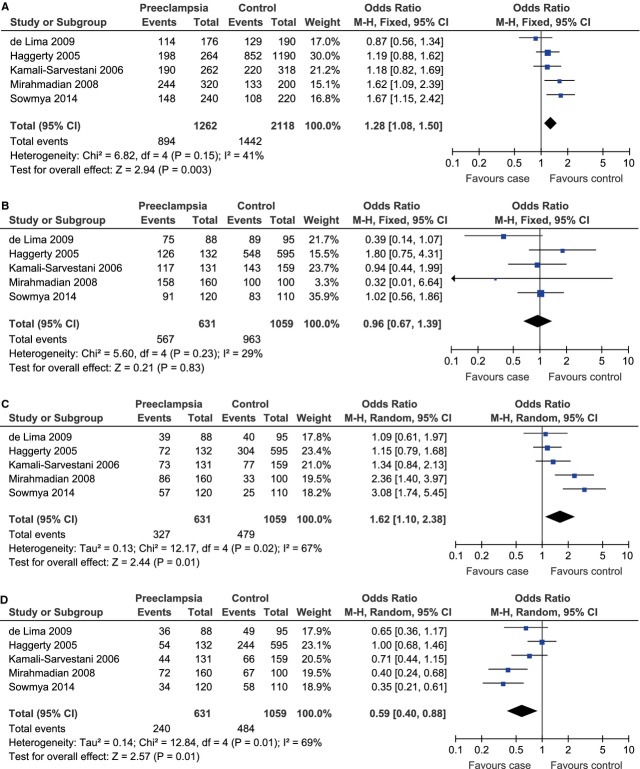
Forest plot of preeclampsia associated with IL-10 -819C/T polymorphism under the allelic (C allele *versus* T allele) (**A**), dominant (CC+CT *versus* TT) (**B**), recessive (CC *versus* CT+TT) (**C**) and co-dominant (CT *versus* CC+TT) (**D**) model.

A total of 631 cases and 1059 controls from 5 case–control studies were included for data synthesis. Under the allelic model, there was no evidence of between-study heterogeneity and therefore the fixed-effect model was adopted to pool the results (C allele *versus* T allele, *P*_heterogeneity_ = 0.15, *I*^2^ = 41%). The meta-analysis results showed that the C allele was associated to the risk of preeclampsia under the allelic model (C allele *versus* T allele, OR = 1.28, 95% CI = 1.08–1.50, *P* = 0.003; Fig. 6A).

As significant between-study heterogeneity was absent under the dominant model (CC+CT *versus* TT, *P*_heterogeneity_ = 0.23, *I*^2^ = 29%) and existed under the recessive (CC *versus* CT+TT, *P*_heterogeneity_ = 0.02, *I*^2^ = 67%) and co-dominant (CT *versus* CC+TT, *P*_heterogeneity_ = 0.01, *I*^2^ = 69%) models, the fixed-effect model was used for the dominant model and the random-effect model was used for the other two genetic models. Under the dominant model, the CC and CT genotypes were not significantly associated with the risk of preeclampsia (CC+CT *versus* TT, OR = 0.98, 95% CI = 0.67–1.39, *P* = 0.83; Fig. 6B). The CC genotype was linked to the risk of preeclampsia under the recessive model (CC *versus* CT+TT, OR = 1.62, 95% CI = 1.10–2.38, *P* = 0.01; Fig. 6C) and the CT genotype did not contribute to the risk of preeclampsia under the co-dominant model (CT *versus* CC+TT, OR = 0.59, 95% CI = 0.40–0.88, *P* = 0.01; Fig. 6D).

### Association between IL-10 -592C/A polymorphism and preeclampsia

**Fig. 7:**
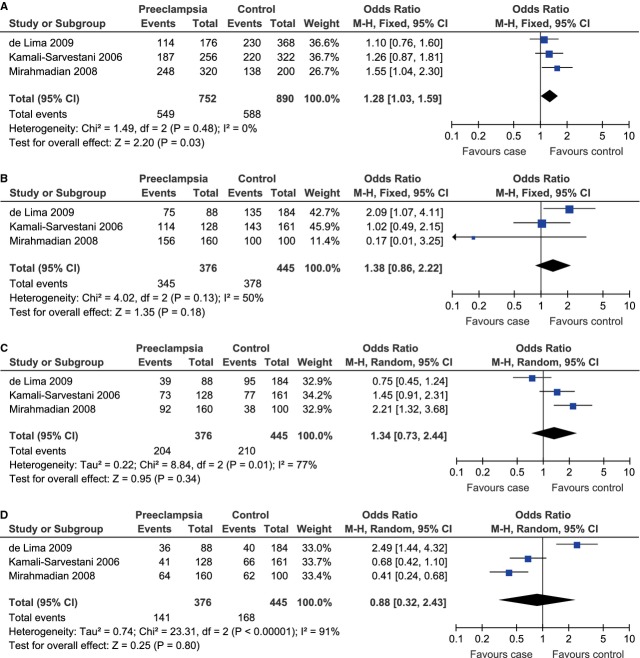
Forest plot of preeclampsia associated with IL-10 -592C/A polymorphism under the allelic (C allele *versus* A allele) (**A**), dominant (CC+CA *versus* AA) (**B**), recessive (CC *versus* CA+AA) (**C**) and co-dominant (CA *versus* CC+AA) (**D**) model.

A total of 376 cases and 445 controls from 3 case–control studies were included for data synthesis. Under the allelic model, there was no evidence of between-study heterogeneity and therefore the fixed-effect model was adopted to pool the results (C allele *versus* A allele, *P*_heterogeneity_ = 0.48, *I*^2^ = 0%). The pooled data demonstrated that the C allele was associated to the risk of preeclampsia under the allelic model (C allele *versus* A allele, OR = 1.28, 95% CI = 1.03–1.59, *P* = 0.03; Fig. 7A).

As significant between-study heterogeneity was absent under the dominant model (CC+CA *versus* AA, *P*_heterogeneity_ = 0.13, *I*^2^ = 50%) and existed under the recessive (CC *versus* CA+AA, *P*_heterogeneity_ = 0.01, *I*^2^ = 77%) and co-dominant (CA *versus* CC+AA, *P*_heterogeneity_ < 0.00001, *I*^2^ = 91%) models, the fixed-effect model was used for the dominant model and the random-effect model was used for the other two genetic models. There was no evidence for any significant association under the dominant (CC+CA *versus* AA, OR = 1.38, 95% CI = 0.86–2.22, *P* = 0.18; Fig. 7B), recessive (CC *versus* CA+AA, OR = 1.34, 95% CI = 0.73–2.44, *P* = 0.34; Fig. 7C) or co-dominant (CA *versus* CC+AA, OR = 0.88, 95% CI = 0.32–2.43, *P* = 0.80; Fig. 7D) model.

### Publication bias

**Fig. 8:**
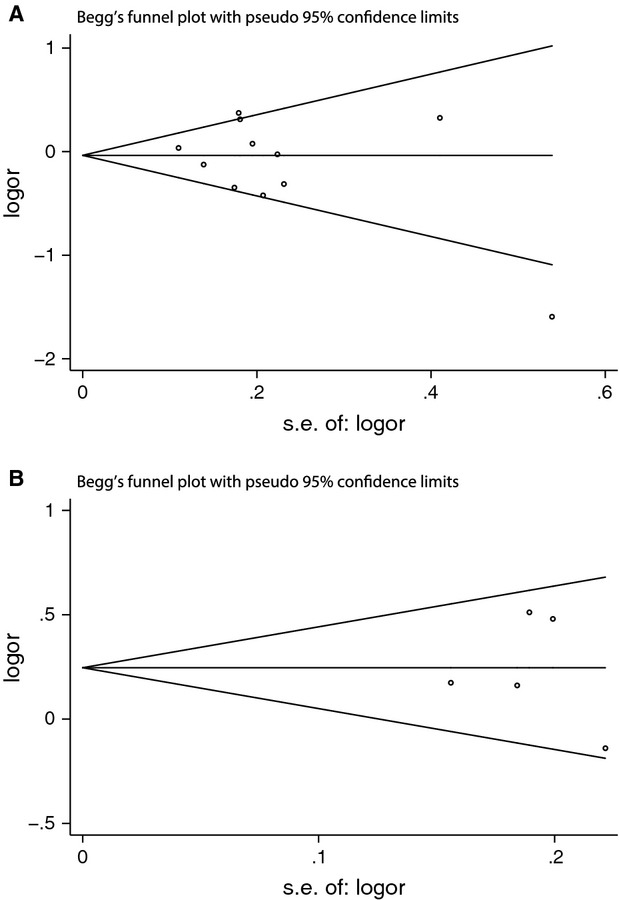
Begger's funnel plot of the meta-analysis of preeclampsia associated with IL-10 -1082A/G (**A**) and -819C/T (**B**) polymorphisms under the allelic model. Each point represents an individual study. Logor: natural logarithm of OR; horizontal line: mean magnitude of the effect; s.e.: standard error.

We used Begg's funnel plot and Egger's linear regression test to evaluate the potential publication bias of the included studies. We did not observe obvious asymmetry of the funnel plot under the allelic model (-1082A/G, *P* = 0.276; -819C/T, *P* = 1.000; Fig. 8). Egger's linear regression test did not show any significant statistical evidence of publication bias under the allelic model (-1082A/G, *P* = 0.307; -819C/T, *P* = 0.844), either. Therefore, no evident publication bias existed in this meta-analysis.

## Discussion

Although the pathogenesis of preeclampsia remains poorly understood, the immunological system is thought to play a pivotal role [43]. It is well know that IL-10 can exert a regulatory effect on Th1/Th2 balance and is a crucial cytokine for pregnancy and development [12,13]. Given the great importance of IL-10 during pregnancy and the influence of its several polymorphic sites in the regulatory regions on its expression levels, several case–control studies have investigated the association between IL-10 gene polymorphisms and the risk of preeclampsia. In the present study, we investigated the association of three maternal IL-10 polymorphisms with preeclampsia.

Interleukin-10 -1082A/G polymorphism was most extensively investigated and there have been three relevant meta-analyses [31–33]. Consistent with these three previous reports, we found that IL-10 -1082A/G polymorphism was not associated with the risk of preeclampsia after including those new relevant studies published between 2011 and 2013. As there existed significant between-study heterogeneity, we performed the subgroup analysis to explore its source. When stratified by continents, the heterogeneity was greatly diminished. Therefore, geographical region was probably a critical factor in between-study heterogeneity and should be better matched between individual studies to further reduce heterogeneity. As Daher *et al*. have reported that the association between IL-10 -1082A/G polymorphism and preeclampsia could be influenced by ethnicity and was only observed in white women instead of non-white women in their study, we analysed such association among white women using four previously published relevant studies and the meta-analysis result did not support the existence of such association among white women. The lack of such association among white women might also be because of the fact that the included studies were from different geographical regions. Besides, we also observed that IL-10 -1082G allele was correlated with the risk of preeclampsia in the Asia and the South American subgroups and both related meta-analysis results favoured the dominant model. Similar association was not observed in the subgroups of Europe and North America. However, meta-analysis could not be performed with only one relevant study available in the subgroups of Africa and Europe-Asia. Therefore, more studies are still called upon to further evaluate the association between IL-10 -1082A/G polymorphism and preeclampsia among persons from different geographical regions.

Both the -819 C allele and the -592 C allele of IL-10 showed evident association with the risk of preeclampsia in our meta-analysis. As these two alleles were related to higher expression levels of IL-10 [30,44] and IL-10 has been thought to facilitate successful pregnancy with IL-10 deficiency leading to preeclampsia [12,24,25], such correlations were somewhat counterintuitive. However, our findings were consistent with the latest meta-analysis result of the association between IL-10 expression level and preeclampsia, which also indicated the elevated IL-10 levels in women with preeclampsia [45]. Although higher IL-10 expression levels have been proposed to provide protection against preeclampsia, further experiments are badly needed to better elucidate the underlying mechanism [6,46]. Besides, our meta-analysis results supported the recessive model for IL-10 -819C/T polymorphism. The association between IL-10 -592C/A polymorphism and preeclampsia was not observed in the dominant, recessive or co-dominant model and probably more studies are needed to investigate the way this polymorphism affects the predisposition to preeclampsia. de Groot *et al*. have reported that IL-10 -2849G/A polymorphism site was also associated with the risk of preeclampsia [42]. However, no other similar studies of such association have been reported. More relevant studies are needed to perform corresponding meta-analyses and confirm such association.

We must admit that there are several limitations in the present study. The total number of the included studies was still relatively small, especially for the meta-analysis of IL-10 -819C/T and -592C/A polymorphisms' association with preeclampsia. The same problem also existed in the subgroup analysis of IL-10 -1082A/G polymorphism's association with preeclampsia stratified by continents. More studies are needed to investigate the role of geographical regions in determining the association between IL-10 -1082A/G polymorphism and preeclampsia. Moreover, other clinical factors like age, gestational weeks and subtypes of preeclampsia (early-onset, late-onset or complicated by other diseases, *etc*.) may result in bias. Further investigation is called upon to determine if these factors affect the results of our meta-analysis.

In conclusion, our meta-analysis results suggested that IL-10 -819C/T and -592C/A polymorphisms were associated with the risk of preeclampsia. Although IL-10 -1082A/G polymorphism had no obvious association with preeclampsia in the overall meta-analysis, it showed association with preeclampsia among Asian and South American populations. However, further studies are essential to validate the association between IL-10 polymorphisms and the risk of preeclampsia.
